# ANKLE ARTHRODESIS WITH INTRAMEDULLARY RETROGRADE NAIL FOR BONE TUMORS. PRELIMINARY RESULTS AND SURGICAL TECHNIQUE

**DOI:** 10.1590/1413-785220233102e264305

**Published:** 2023-06-09

**Authors:** JOSÉ MANUEL CASANOVA, JOÃO PAULO FREITAS, RUBEN LOPES FONSECA, PAULO TAVARES, DIOGO LINO MOURA

**Affiliations:** 1. Orthopedic Oncology Department, Centro Hospitalar e Universitário de Coimbra, Coimbra, Portugal.; 2. Faculty of Medicine, Universidade de Coimbra, Coimbra, Portugal.

**Keywords:** Tumors, Ankle, Intramedullary nailing, Neoplasms, bone tissue, Giant cell tumors, Osteosarcoma, Neoplasias, Tornozelo, Fixação intramedular de fraturas, Neoplasias de tecido ósseo, Tumores de células gigantes, Osteossarcoma

## Abstract

**Objective:**

Present the preliminary results of a case series using the surgical ankle arthrodesis technique with an intramedullary retrograde nail for bone tumors.

**Methods:**

We present the preliminary data of 4 patients, 3 males and 1 female, with a mean age of 46,2 (range 32 to 58) years, with histology proven Giant Cell Tumour of bone in 3 and osteosarcoma in 1. The mean resection length of distal tibia was 11,75 (range 9 to 16) cm, and all the patients underwent reconstruction with a tibiotalocalcaneal arthrodesis with an intercalary allograft fixed by a retrograde intramedullary nail.

**Results:**

Oncological follow-up evolved without evidence of local recurrence or disease progression in all patients. After a mean time of 69.5 (range 32 to 98 months), patients had a mean MSTS12 functional score of 82.5% (range 75 to 90). All tibial arthrodesis and diaphyseal osteotomy sites were fused within 6 months with a return to activities without complications related to coverage skin or infection.

**Conclusion:**

No complications were recorded; all arthrodesis and diaphysial tibial osteotomy sites fused by 6 months, and the mean follow-up of those patients was 69,5 (range 32 to 988) months, with a mean functional MSTS score of 82,5% (range 75-90). Level of Evidence: IV; Retrospective Case Series.

## RESUMO

**Objetivo:**

Apresentar os resultados preliminares de uma série de casos utilizando a técnica cirúrgica de artrodese do tornozelo com haste intramedular retrógada para tumores ósseos.

**Métodos:**

Apresentamos os dados preliminares de quatro pacientes, três homens e uma mulher, com idade média de 46,2 (variação de 32 a 58) anos, com histologia comprovada de tumor de células gigantes em três e osteossarcoma em um. O comprimento médio de ressecção da tíbia distal foi de 11,75 (variação de 9 a 16) cm, e todos os pacientes foram submetidos à reconstrução com uma artrodese tibiotalocalcaneana com um aloenxerto intercalar fixado por uma haste intramedular retrógrada.

**Resultados:**

O acompanhamento oncológico evoluiu sem evidências de recidiva local ou progressão da doença, em todos os pacientes. Após um tempo médio de 69,5 (variação de 32 a 98 meses), os pacientes tiveram uma pontuação média funcional MSTS12 de 82,5% (variação de 75 a 90). Todos os locais de artrodese e osteotomia diafisária tibiais foram fundidos em 6 meses com retorno às atividades de vida diária sem complicações relacionadas à cobertura ou infecção.

**Conclusão:**

Não foram registradas complicações; todos os locais de artrodese e osteotomia diafisária da tíbia fundiram-se em 6 meses, e o acompanhamento médio desses pacientes foi de 69,5 (variação de 32 a 988) meses, com uma pontuação média funcional MSTS de 82,5% (variação de 75-90). Nível de Evidência IV; Série de Casos Retrospectivos.

## INTRODUCTION

For a long period of time, those rare tumours located at the distal tibia, were candidates for amputation, mostly due to the poor soft tissue coverage. With the development of more effective arthrodesis surgical techniques, the use of chemotherapy in malignant tumours, and the need to save the limb in tumours of a benign nature, and also in patients that refused amputation, limb salvage surgery has become a demand for the majority of situations. Reconstructive options in distal tibia tumours include the use of a custom made prosthesis, a modular prosthesis, osteoarticular allografts and ankle arthrodesis by the use of free or vascularized bone grafts. The fixation for the arthrodesis is variable, from antegrade nailing, plates and more recently with retrograde intramedullary nails.

A few small series have been reported in the literature dealing with this tumour location and the particular needs for reconstruction.^1 - 9^

## PATIENTS AND METHODS

Medical records, operative reports, radiographs and histologic records of all patients included in the study were reviewed.

From 2007 to 2013, four patients with distal tibia bone tumours, were submitted to tumor resection and reconstruction by ankle arthrodesis with intercalary allograft and fixation with a retrograde transtalar intramedullary nail.

There were 3 males and 1 female, with a mean age at diagnosis of 46,2 (range 32 to 58) years. 3 patients had a benign bone tumour (giant cell tumour), all of which were Stage 3 (according with the Enneking Staging System)^10^ and one patient had a malignant bone tumour (osteosarcoma) Stage IIB, using the same Staging System.

This patient with high grade malignant osteosarcoma, was treated with neoadjuvant and adjuvant chemotherapy, with a four drug regimen composed by Methotrexate; Adriamycin, Ifosfamide and Cisplatin, and obtained a Huvos Grade IV response^11^ after neoadjuvant chemotherapy.

All patients underwent surgical treatment, with a tumour resection with wide margins in the case of the sarcoma, and marginal in the benign aggressive GCT. A sample from the bone marrow of the proximal side of the tibia osteotomy stump, was obtained, and evaluated by frozen section, to access margins adequacy, and to avoid spread of the disease during nail insertion. In the 4 patients an ankle arthrodesis was obtained using an intercalary allograft, fixed with a retrograde intramedullary tibiotalocalcaneal nail and an additional proximal osteotomy site fixation with an anti-rotation plate, and surround filling of the osteotomy with autologous bone chips from the iliac crest.

After suture removal, the patients were immobilized with a cast for 4 weeks without weight bearing. After four weeks the plaster was changed to a delta cast with the application of a walking shoe sole, and progressive weight bearing was allowed, until full weight bearing is reached around ten weeks post-operatively. The patients remain in the cast for 6 months and walk with full weight bearing with the support of a cane.

Clinical and radiograph follow-up was obtained every month. ( Table 1 )

**Table 1:** Patients data.

Patient	Age (years) Gender	Diagnosis, stage, margins	Length of tibial resection (cm)	Bone grafts	Fixation technique	Complications	Follow-up (m)	Functional scores -MSTS/ISOLS
1	58 Male	GCT 3 Marginal	9	Allograft + Autologous bone chips from iliac crest	Retrograde intramedullary nail + antirotational plate and screws	No	98	80
2	40 Male	GCT 3 Marginal	12	Allograft + Autologous bone chips from iliac crest	Retrograde intramedullary nail + antirotational plate and screws	No	86	85
3	32 Male	Osteosarcoma IIB Wide	16 (fibula 12)	Allograft + Autologous bone chips from iliac crest	Retrograde intramedullary nail + antirotational plate and screws	No	62	90
4	55 Female	GCT 3 Marginal	10	Allograft + Autologous bone chips from iliac crest	Retrograde intramedullary nail + antirotational plate and screws	No	32	75

### Surgical technique

At the start of the surgical procedure autologous bone chips are harvested from the iliac crest.

One of the major difficulties after distal tibia resection is to obtain correct alignment for the ankle and foot while doing the arthrodesis. This difficulty is potentiated by the gap space at the distal tibia in the resection location, which favours additional movement, sometimes without proper control during the process of nailing.

**Figure 1:**
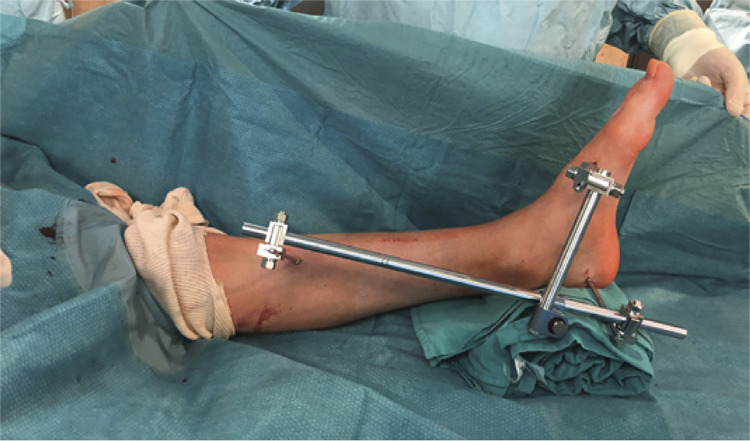
External fixator in place, keeping the length and position of the foot during surgery.

To avoid malalignment of the ankle and foot during the arthrodesis process and also to eliminate difficulties at the selection of the nail entry point in the calcaneus avoiding a fracture, the next surgical step is to mount an external tubular fixator, putting a Chance pin at the foremost desirable position at the proximal tibia, another pin at the posterior tip of the calcaneus, and a third pin at the third metatarsal bone to position the foot. ( Figure 1 )

That way the foot and ankle can be maintained in a proper position for the arthrodesis – with ankle neutral dorsiflexion, 5 degrees heel valgus and external rotation of the foot in relation to the tibia comparable to that of the normal contralateral side, of approximately 5 to 10 degrees. Also under fluoroscopy the relation between the calcaneus and the tibia, can be confirmed, allowing the proper valgus inclination that will permit the correct nailing positioning because by this method the normal lateral offset of the calcaneus in relation with the tibia is corrected.^13^

The positioning of the external fixator is applied in a way that does not make the surgical resection any more difficult.

In all patients surgery was performed using an anterior approach to the ankle and in osteosarcoma case a lateral approach was added to facilitate the distal fibula resection together in the same surgical specimen with the distal tibia where the tumour had origin.

The average resection length of distal tibia was 11,75 (range 9 to 16) cm, and the tibial osteotomy was performed at least 3 cm proximal to the tumour extension as determined by preoperative MRI.

Following tumour resection the defects were reconstructed with a tibia allograft selected previously from our Bone Bank, matching the dimensions of the resected bone based on the preoperative planning scans. An allograft that is slightly undersized than the host bone is preferred but the most important match is at the proximal osteotomy site.

**Figure 2:**
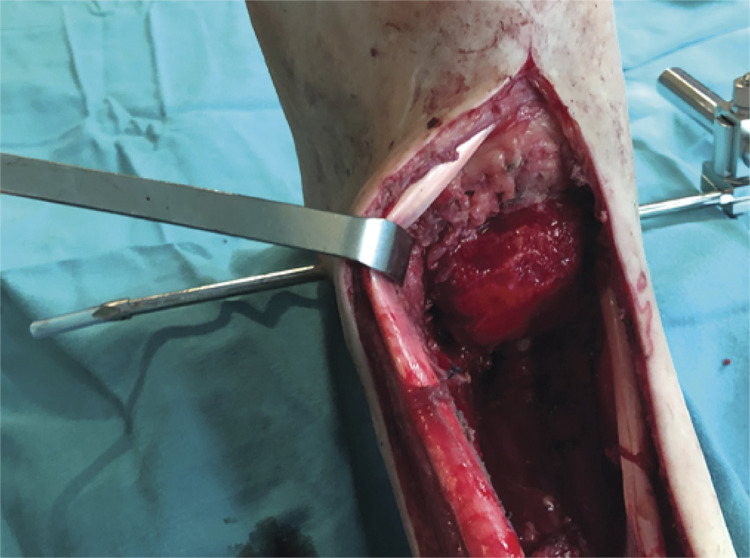
Talus prepared to receive the allograft. Notice the removal of cartilage, the adaptation of the dome to the allograft size, and the design of a groove to receive the allograft extremity.

The articular cartilage of the dome of the talus is removed with a high speed burr, after which an elliptical tip burr is used to carefully we carefully design around the talus dome a groove where the distal part of the allograft is to be seated. ( Figure 2 ) If needed additional autologous bone chips, previously harvested from the iliac crest can be used.

This way an ankle arthrodesis is obtained using an allograft in an intercalary position.

After all these steps are achieved, a retrograde intramedullary nail is inserted from bellow checking the calcaneotalotibial progression under fluoroscopy control, in a way that when the tip of the nail abuts the talus dome, there is visual control of the position.

In the next surgical step, and now that the correct arthrodesis position of the foot and ankle is assured, distraction of the surgical gap is done, using the external fixator to obtain it, and the allograft, is carefully positioned in place, modifying its size if necessary. With the alignment been maintained by the external fixator, the graft is now compressed again using the external fixator^14^ , and then the nail is progressed proximally through the interior of the allograft and after inside the host proximal tibial bone. With this surgical technique, the correct position for the arthrodesis is assured as well as the correct placement of the allograft in the most anatomic position, and avoiding possible allograft fracture during the progression of the nail.

Because of the length of resections, straight nails, between 200 and 300 mm, are used depending on the resection length.

**Figure 3:**
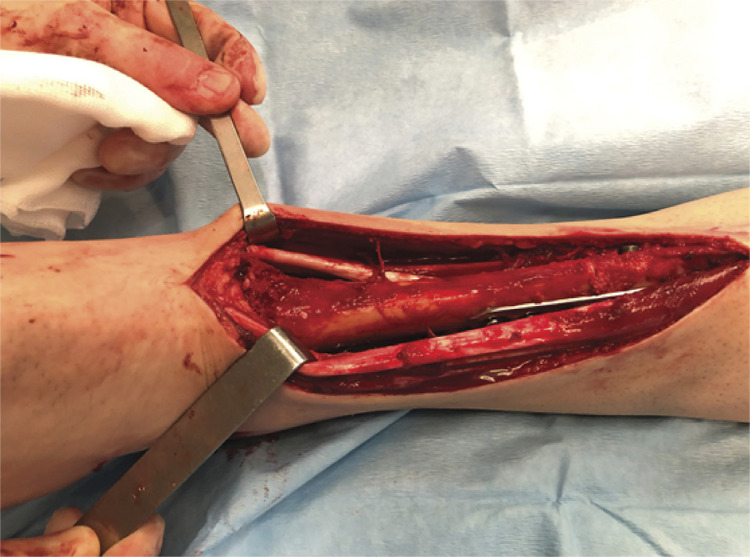
Image after the procedure final, before closing. (Transtalar nail insertion, external fixator removal, foot positioning correction, passage of the proper screws through the nail, autologous grafting and antirotation plating).

As a final step at the proximal host-allograft bone osteotomy site, an antirotational plate, is placed laterally, also compressing the osteotomy site, supported by cortical screws, and the cancellous autografts harvested from the iliac crest are then placed around the osteotomy site. ( Figure 3 )

Surgery is finalized with a careful suturing and soft tissue coverage under a suction drainage system. Good soft tissue coverage is pivotal, and if primary wound closure could not be achieved then flaps coverage may be required, although it was not needed in this group of patients. An antalgic posterior cast is than applied.

## RESULTS

Oncological follow-up progressed with no evidence of local recurrence or disease progression, for all patients. At last follow-up after a mean time of 69,5 (range 32 to 98) months, the patients had a mean functional MSTS^12^ score of 82,5% (range 75-90) at last follow-up. All arthrodesis and diaphysial tibial osteotomy sites fused by 6 months. All patients have returned to their previous professions, all of them walk without canes and one of them swims regularly. There were no complications related with skin coverage, wound breakdown or infection.

## DISCUSSION

After distal tibia resections for bone tumours different reconstructive options have been reported.

The use of a large prosthesis in the ankle joint has a high risk for septic and mechanical complications, mostly late, like prosthetic loosening or talar collapse, that contribute to increasing functional disability over time. These complications are also known from the literature, related with the use of prosthetic replacements even in degenerative pathologies. This evidence can be based on Abudu et al,^2^ Lee et al^15^ and Shekkeris et al^8^ series where an early functional recovery was reported, but significant midterm complications including deep infections and aseptic loosening arose. With time the function continues to deteriorate with growing disability, discomfort and pain.

Massive osteoarticular allografts are not commonly used at this location, mainly because of joint instability and allograft fracture often associated with implant failure.^1^

Because of the previous reasons the most used reconstructive technique for limb salvage surgery of the distal tibia is ankle arthrodesis, because it provides a stable joint and residual satisfactory function.

Reconstruction techniques to achieve ankle arthrodesis described after distal tibia tumour resection include different and variable types of grafts and fixation materials.

Vascularized autogenous fibula or iliac crest grafts, autogenous and/or allograft cortical structural grafts, were used by different authors, who also using different types of fixation, varying from Illizarov external fixators to plates or antegrade nails.^1 , 3 , 4 , 6 , 7^

Moore et al^5^ reported on 9 patients with resections followed by reconstruction with ankle arthrodesis using a massive allograft and retrograde nail fixation, with a major complication rate of 44,4% and an MSTS mean score of 73.

Recently Xu et al^9^ reported on a series of 5 patients with a high grade malignant osteosarcoma of the distal tibia, in whose reconstruction an allograft and a retrograde intramedullary nail was used, with good functional results and few complications.

Dieckmann^16^ et all, firstly described a tibiotalocalcaneal arthrodesis with a retrograde nail, for the surgical treatment of malignant tumours with resection of the entire fibula.

Table 2 summarizes the available literature reports.

**Table 2:** Summary of the literature based on published series.

Author Publication year	Number of cases	Reconstruction	Complications	ISOLS/MSTS score	Follow-up Years
Abudu ^1^ 1999	5	Custom-made semiconstrained rotating hinge prosthesis	Deep infection (1) Early aseptic loosening of talar component (1) Local recurrence (1)	Initial 1 y – 81 Decreased in time for 50	5,5
Lee ^14^ 1999	6	Custom-made hinged prosthesis	Infection (1) Talar collapse (1)	80,5	5,3
Shekkeris 2009^8^	6	Endoprosthetic replacement	Infection 2 (lead to amputation)	70%	9.6
Engelhardt and Morant ^15^ 1993 Taylor^7^ 2003	1 1	Illizarov bone transportation	-	-	6
Bishop ^3^ 1995	4 tumor cases 7 trauma/osteomyelitis	<4 cm defect – iliac crest vascularized graft >4 cm defect vascularized fibula	Deep infection (1) Non Union (1) (amputations)	-	-
Shalaby ^6^ 2006	6	Autogenous fibular strut graft (vascularized in 3) + cancelous autogenous graft Illizarov fixation	New grafting (2) Local recurrence (1)	70	-
Moore ^5^ 2005	9	Massive allograft and retrograde nail fixation	Fractures (3) Non union (1) Mechanical complications (3) 65,4% rate new surgery	73	-
Xu ^9^ 2017	5	Allograft and retrograde nail fixation	Subcutaneous fluid in 4	74,3%	3,6
Casadei ^4^ 1994	12	Different reconstructions: Autografts Autografts + cortical allografts Vascularized fibula Fixation with Kuntcher or Grosse-Kempf nail/or plates	Deep infection (2) Graft Fractures (4)	-	5,6
Campanacci ^1^ 2008	8	Cortical structural allografts and/or autogenous grafts. <8 cm defect – autografts >8 cm defect auto + allografts Fixation: Antegrade nail 6 Plate 2	Donor site (tibia): Infection (1) Fracture (1) Deep infection (1) – amputation Local recurrence (1)	80,4	4,5

What can be pulled out of that table, is the following: 1) The published series report on a few number of cases 2) Even the series that present a larger number of cases, the surgical reconstruction methods were different in the same series, which can bias the results. 3) Although arthrodesis is a function-limiting procedure, a MSTS score greater than 70 was reported in the majority of the series. 4) For the same series good results, in terms of pain, support-free walking and emotional acceptance, were achieved.^17^

**Figure 4:**
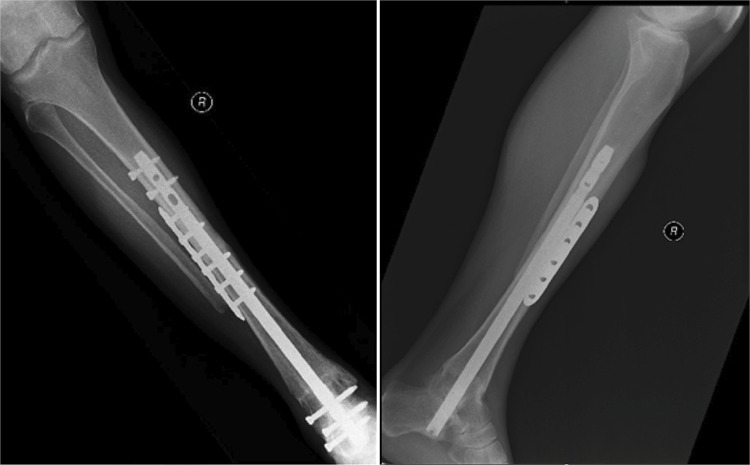
Post operative XR at 10 months post-operative, with allograft incorporation on both osteotomies sites.

One of the issues when using a retrograde nail is that it’s use results in the sacrifice of the subtalar joints, when its involvement in the tumour is not present, and a need to extend the arthrodesis to that joint is oncologically unjustified. We strongly think, that although not necessary in relation to an oncology point of view, the possibility of secondary arthritic changes in adjacent foot joints that occur, with time, due to the loss of tibiotalar motion is quite significant, and more frequently found in the subtalar joints.^18^ The sacrifice of the subtalar joints supressing the motion also in this joint, decreases significantly the pain and increases the mechanical support of the arthrodesis, contributing also for the short period that the distal calcaneotalofibular fusion demands to occur (4 to 6 months), and with the achievement of that distal fusion, allowing stability for a quicker proximal osteotomy fusion. ( Figure 4 )

## CONCLUSION

In our experience, even in this small series, in cases of distal tibia tumours, ankle arthrodesis using an intercalary allograft, fixed by a retrograde intramedullary nail is an efficient method of limb reconstruction after tumor resection. The surgical technique described, led to an absence of complications and successful fusion in every case with good functional and oncological outcomes.
